# 

**Published:** 1865-06

**Authors:** 


### BOOKS AND PAMPHLETS RECEIVED.

*Physicians' Prescription Book; containing lists of the terms, phrases, contractions, and abbreviations used in prescriptions, with explanatory notes; the grammatical construction of prescriptions; rules for the pronunciation of Pharmaceutical terms; a prosodiacal vocabulary of the names of drugs, etc.; and a series of abbreviated prescriptions illustrating the use of the preceding terms. To which is added a Key, containing the Prescriptions in an unabreviated form, with a literal translation, for the use of Medical and Pharmaceutical students.* By Jonathan Pereira, M. D., F. R. S. *Fourteenth edition. Philadelphia:* Lindsay & Blakiston, 1865.

*The Renewal of Life—Lectures, chiefly Clinical.* By Thomas King Chambers, M. D., *Honorary Physician to H. R. H. the Prince of Wales; Physician to St. Mary's Hospital. From the third London edition. Philadelphia:* Lindsay & Blakiston, 1865.

*Annual Report of the City Inspector of the City of New York, for the year ending December* 31, 1864. *Document No.* 7.

*The Ophthalmic Review; a Quarterly Journal of Ophthalmic Surgery and Science. Edited by* J. Zachariah Laurence *of London, and* Thomas Windsor *of Manchester, Eng. April number.*

*Contributions to Practical Surgery.* BY W. H. VAN BUREN, M. D., *Professor of Anatomy, University of New York; formerly one of the Surgeons of the New York Hospital, of the Bellevue Hospital, and Vice President of the N. Y. Academy of Medicine; consulting Surgeon to St. Vincent's Hospital, and the Woman's Hospital; member of the Pathological Society and the Medical and Surgical Society of New York; member of the U. S. Sanitary Commission, etc., etc. Philadelphia:* J. B. LIPPINCOTT & CO., 1865.

*Household Poems.* BY HENRY W. LONGFELLOW; *with illustrations by* JOHN GILBERT, BIRKET FOSTER, AND JOHN ABSOLON. *Boston:* TICKNOR & FIELDS, 1865.

*Catalogue and Circular of the Albany Medical College,* 1864.

*The Army Surgeon's Manual.* BY WILTIAM GRACE, *of Washington, D. C. Second edition. New York:* BAILLIERE BROTHERS.

*Receipt of Music.*—We have to acknowledge the receipt of sheets of some very fine music—"Funeral March to the Memory of Abraham Lincoln," and "Oh, send me one Flower from his Grave." Published by Horace Waters, 181 Broadway, New York.

